# A network pharmacology approach to reveal the protective mechanism of *Salvia miltiorrhiza*-*Dalbergia odorifera* coupled-herbs on coronary heart disease

**DOI:** 10.1038/s41598-019-56050-5

**Published:** 2019-12-18

**Authors:** Fei Li, Jialin Duan, Meina Zhao, Shaojie Huang, Fei Mu, Jing Su, Kedi Liu, Yang Pan, Xinming Lu, Jing Li, Peifeng Wei, Miaomiao Xi, Aidong Wen

**Affiliations:** 1 Department of Pharmacy, Xijing Hospital, Fourth Military Medical University, Xi’an, Shaanxi 710032 China; 2 Department of Pharmacy, The Hospital of 92012 Troops, PLA Navy, Zhoushan, Zhejiang 316000 China; 3 Department of Chinese Materia Medical and Natural Medicines, School of Pharmacy, Fourth Military Medical University, Xi’an, Shaanxi 710032 China; 4 College of Pharmacy, Shaanxi University of Chinese Medicine, Xianyang, Shaanxi 712046 China; 5 TANK Medicinal Biology Institute of Xi’an, Xi’an, Shaanxi 710032 China; 6 YouYi Clinical Laboratories of Shaanxi, Xi’an, Shaanxi 710032 China

**Keywords:** Protein-protein interaction networks, Virtual drug screening, Protein-protein interaction networks, Cardiovascular biology, Cardiovascular biology

## Abstract

*Salvia miltiorrhiza*-*Dalbergia odorifera* coupled-herbs (SMDOCH) has been used to treat coronary heart disease (CHD) for thousands of years, but its unclear bioactive components and mechanisms greatly limit its clinical application. In this study, for the first time, we used network pharmacology to elucidate the mechanisms of action of SMDOCH on CHD. We collected 270 SMDOCH-related targets from 74 bioactive components and 375 CHD-related targets, with 58 overlapping common targets. Next, we performed enrichment analysis for common-target network and protein-protein interaction (PPI) network. The results showed that SMDOCH affected CHD mainly through 10 significant signaling pathways in three biological processes: ‘vascular endothelial function regulation’, ‘inflammatory response’, and ‘lipid metabolism’. Six pathways belonged to the ‘vascular endothelial function regulation’ model, which primarily regulated hormone (renin, angiotensin, oestrogen) activity, and included three key upstream pathways that influence vascular endothelial function, namely KEGG:04933, KEGG:05418, and KEGG:04066. Three pathways, namely KEGG:04668, KEGG:04064, and KEGG:04620, belonged to the ‘inflammatory response’ model. One pathway (KEGG:04920) belonged to the ‘lipid metabolism’ model. To some extent, this study revealed the potential bioactive components and pharmacological mechanisms of SMDOCH on CHD, and provided a new direction for the development of new drugs for the treatment of CHD.

## Introduction

Coronary heart disease (CHD), a leading cause of death worldwide, is characterized by myocardial dysfunction and/or organic lesions caused by insufficient blood and supply coronary artery stenosis^1,2^. Chronic ischemia caused by coronary artery stenosis or myocardial infarction can lead to heart failure and/or death. Ischemic heart failure, angina pectoris, and myocardial infarction are all CHD, which is the main pathogenesis of acute myocardial infarction^3^.

CHD is considered a chest syndrome in Traditional Chinese Medicine (TCM). *Salvia miltiorrhiza*-*Dalbergia odorifera* coupled-herb (SMDOCH), a well-known coupled-herb from the *‘Golden Chamber’, ‘New Qianjinfang’*, and *‘Medical Correction’*, has been used to treat chest syndromes in China, Japan, and South Korea for thousands of years. SMDOCH is the main component in Chinese medicines such as Guanxin II, Danxiang Guanxin Injection, and Danxiang Injection. However, the international promotion and secondary development of SMDOCH are limited because the bioactive components and mechanism of action of SMDOCH are unclear.

TCM formulas exert their effect on multiple biological processes to treat diseases via its diverse bioactive components, which act on multiple targets. However, most studies still use the term ‘single-component’, ‘single-target’, or ‘single-pathway’ to investigate the mechanism of TCM. With the development of bioinformatics, network pharmacology has become a highly effective method for studying TCM, as it can reveal the relationship between the bioactive components of a TCM and their potential mechanism of action in a systematic and comprehensive manner^4^.

To comprehensively elucidate the mechanism of SMDOCH on CHD using network pharmacology, we first screened the targets of SMDOCH bioactive components and CHD-related targets. Next, we constructed a SMDOCH-CHD common-target network and a core-target protein-protein interaction (PPI) network, and then performed a cluster analysis of the core-target PPI network. Finally, we screened the key targets and signaling pathways by GO and KEGG pathway enrichment analysis to further explore the protective mechanism of SMDOCH on CHD.

## Results

### SMDOCH component-target network

**Figure 1:**
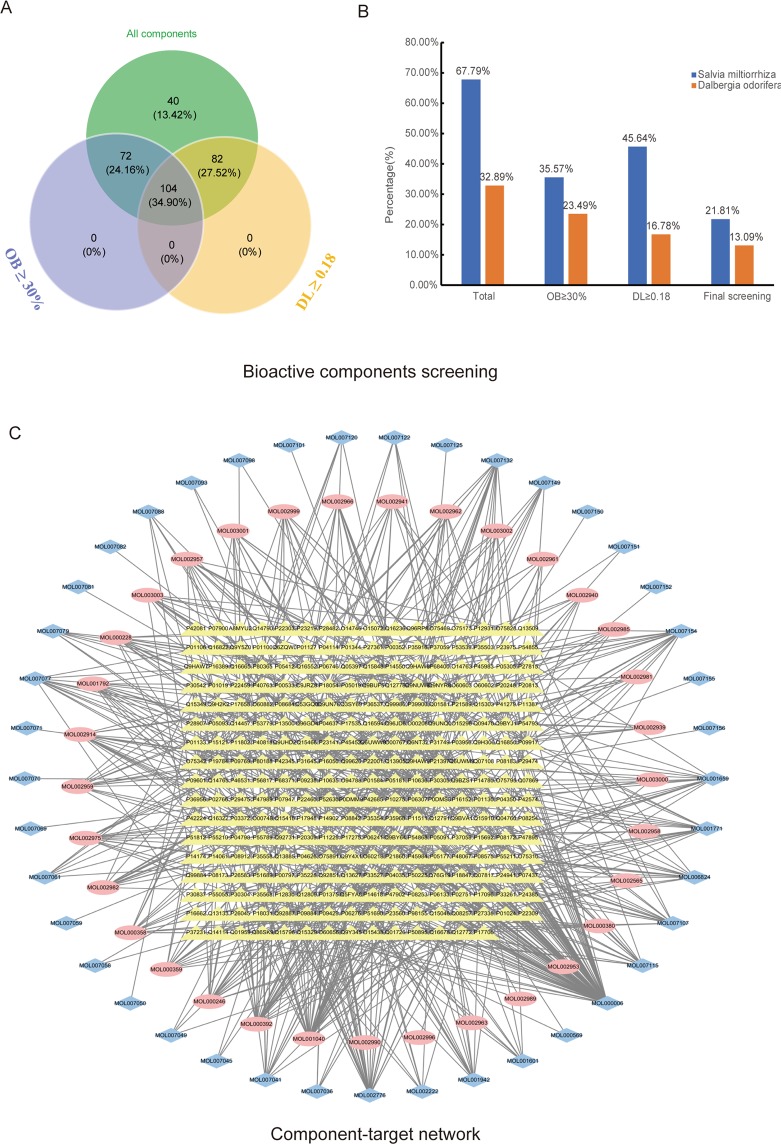
SMDOCH component-target network. (**A**) Venn diagram: 298 components (green section), and 104 bioactive components screened by two ADME-related models (blue section stands for the components of OB ≥ 30%, yellow section stands for DL ≥ 0.18). (**B**) Distributions of different herbs. (**C**) Construction of SMDOCH component-target visual network, including 344 nodes and 691 edges. Blue nodes and pink nodes stand for bioactive components from SM and DO respectively, yellow nodes stand for targets.

A total of 298 components of SMDOCH were collected from two natural product databases: TCMSP and TCM@Taiwan, 202 of which were from *S. miltiorrhiza* (SM), 98 from *D. odorifera* (DO), and 2 (palmitic acid, alpha-Farnesene) from SM and DO. The components were screened with the criteria of OB ≥ 30% and DL ≥ 0.18. In total, 104 bioactive components of SMDOCH were included, 62.5% (65/104) and 37.5% (39/104) of which were from SM and DO, respectively (Fig. 1A,B). Subsequently, we obtained the structural information of the bioactive components, including molecular structures, canonical smiles, and their ‘sdf’ files from the product databases of PubChem and ZINC.

Next, we used a similarity-based method to identify the targets of the 104 bioactive components with the public webservers of Swiss Target Prediction and STITCH. Only 74 of 104 bioactive compounds had effective targets, 41 of which were from SM and the remaining from DO. From the 74 bioactive components, 270 potential targets were explored after removing duplicates. We subsequently constructed a visual SMDOCH component-target network containing 344 nodes and 691 edges by using Cytoscape (Fig. 1C). The number of these targets in SM and DO was 216 and 118 respectively, and 64 targets overlapped in the two herbs, which indicated that SM and DO showed the propensity to interact with each other by acting on the same or similar targets. We then conducted further studies to explore the interaction between SM and DO.

### Common-target network

**Figure 2:**
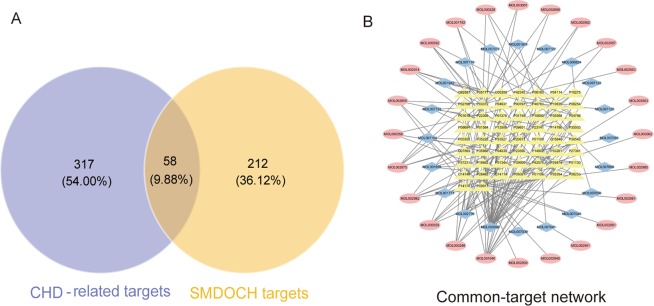
Common-target network. (**A**) 58 targets are common to SMDOCH and CHD. (**B**) Common-target network, including 102 nodes and 145 edges. Blue nodes and pink nodes stand for bioactive components from SM and DO respectively, yellow nodes stand for targets.

The occurrence and development of CHD involves the co-regulation of multiple genes. Investigation of gene and gene-environment interaction is beneficial to elucidate the pathogenesis of CHD. In this study, 375 CHD-related targets were collected from human genomic databases. The number of these targets in OMIM, TTD, NCBI Gene, PharmGkb, Drugbank, CTD, and GeneCards was 248, 30, 73, 27, 31, 48, and 17, respectively. In addition, 58 targets were common to SMDOCH and CHD (Fig. 2A,B), and these targets were related to 20 and 24 bioactive components from SM and DO, respectively. We used degree, a network pharmacological parameter based on topological analysis method that reflects the importance of nodes through the number of connections to other nodes, to screen 18 candidate components with a criteria of degree > 2 in the common-target network. Some of the candidate components, such as tanshinone IIA from SM and formononetin and butin from DO, were highly abundant in SMDOCH and their pharmacological activities will be investigated in future studies.

CHD development is closely related to several biological processes, including vascular tone/nitric oxide signaling, cellular proliferation/vascular remodelling, and inflammation/cell adhesion/transendothelial migration, as well as molecules such as Low Density Lipoprotein (LDL) cholesterol and triglyceride-rich lipoproteins^5^. These pathogenesis-related biological processes and molecules mainly involve three functional modules, including vascular endothelial function regulation (vascular tone/nitric oxide signaling, cellular proliferation/vascular remodelling), inflammatory response (inflammation/cell adhesion/transendothelial migration), and lipid metabolism (LDL cholesterol and triglyceride-rich lipoproteins). To elucidate the mechanism of SMDOCH in the treatment of CHD, the 58 common targets were input into DAVID and ClueGO for GO and KEGG pathway analysis, producing 35 biological processes (BPs), 7 cell components (CCs), 13 molecular functions (MFs), and 93 KEGG pathways.

According to the aetiologies of CHD, these biological processes can be divided into three functional modules, including vascular endothelial function regulation (GO:0045429, GO:1904707, GO:0001666, GO:0071456, GO:0043066, KEGG:04933, KEGG:05418, KEGG:04915, KEGG:04066, KEGG:04370, KEGG:04210 and KEGG:04924), inflammatory response (GO:0031663, GO:0032496, GO:0071260, KEGG:04668, KEGG:04064 and KEGG:04620), and lipid metabolism (GO:0034383, KEGG:04920, KEGG:04152, KEGG:03320 and KEGG:04976) (Table 1).

**Table 1:** Functions of 58 common targets based on GO and KEGG pathway analysis through DAVID and ClueGO.

Classification	ID	Term
Vascular endothelial function regulation	GO:0045429	Positive regulation of nitric oxide biosynthesis
	GO:1904707	Positive regulation of vascular smooth muscle cell proliferation
	GO:0001666	Response to hypoxia
	GO:0071456	Cellular response to hypoxia
	GO:0043066	Negative regulation of apoptotic process
	KEGG:04933	AGE-RAGE signaling pathway in diabetic complications
	KEGG:05418	Fluid shear stress and atherosclerosis
	KEGG:04915	Oestrogen signaling pathway
	KEGG:04066	HIF-1 signaling pathway
	KEGG:04370	VEGF signaling pathway
	KEGG:04210	Apoptosis
	KEGG:04924	Renin secretion
Inflammatory response	GO:0031663	Lipopolysaccharide-mediated signaling pathway
	GO:0032496	Response to lipopolysaccharide
	GO:0071260	Cellular response to mechanical stimulus
	KEGG:04668	TNF signaling pathway
	KEGG:04064	NF-κB signaling pathway
	KEGG:04620	Toll-like receptor signaling pathway
Lipid metabolism	GO:0034383	Low-density lipoprotein particle clearance
	KEGG:04920	Adipocytokine signaling pathway
	KEGG:04152	AMPK signaling pathway
	KEGG:03320	PPAR signaling pathway
	KEGG:04976	Bile secretion

### SMDOCH-CHD PPI network

**Figure 3:**
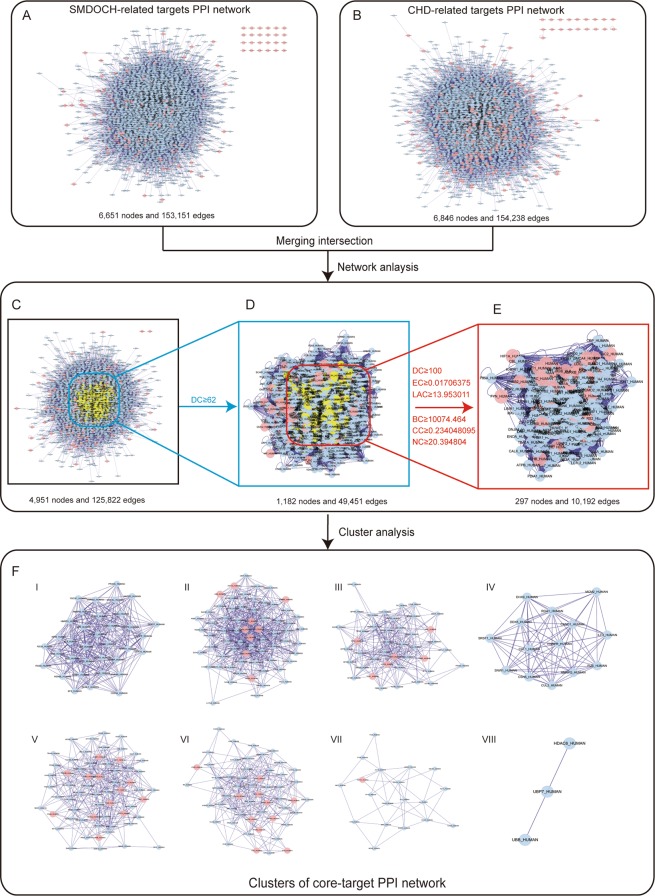
SMDOCH-CHD PPI network. (**A**) SMDOCH-related targets PPI network (6,651 nodes and 153,151 edges). (**B**) CHD-related targets PPI network (6,846 nodes and 154,238 edges). (**C**) Intersection of PPI networks (4,951 nodes and 125,822 edges). (**D**) PPI network by the screening criteria of ‘DC ≥ 62’ (1,182 nodes and 49,451 edges). (**E**) Core-target PPI network by the screening criteria of “‘DC’ ≥ 100, ‘EC’ ≥ 0.01706375, ‘LAC’ ≥ 13.953011, ‘BC’ ≥ 10074.464, ‘CC’ ≥ 0.234048095 and ‘NC’ ≥ 20.394804” (297 nodes and 10,192 edges). (**F**) Clusters of core-target PPI network. Pink nodes stand for SMDOCH-related targets and CHD-related targets, yellow nodes and blue nodes stand for selected targets by the screening criteria and other human proteins respectively.

The PPI networks of SMDOCH- and CHD-related targets were constructed by using Bisogenet (Fig. 3A,B). We selected the same nodes and edges from the two PPI networks to obtain an intersection (Fig. 3C). Subsequently, we used CytoNCA to perform a central network assessment of the intersection of the PPI networks through topological analysis, and determined the significant targets for SMDOCH on CHD PPI network by a screening criteria of ‘DC ≥ 62’ (Fig. 3D). Six screening criteria were used to further screen the network, and a core-target PPI network containing 297 candidate targets for SMDOCH on CHD was finally obtained (Fig. 3E).

To clarify the mechanism of the 297 candidate targets for SMDOCH on CHD, we analysed the core-target PPI network using cluster analysis by MCODE, generating eight clusters (Fig. 3F) that were subsequently analysed. The cluster analysis produced 40 input targets from SMDOCH- or CHD-related targets, 8 of which belong to the 58 common targets, including TP53, ESR1, AKT1, STAT3, MAPK1, AR, MYC, and PPARG. These targets may be the core targets of SMDOCH on CHD.

**Figure 4:**
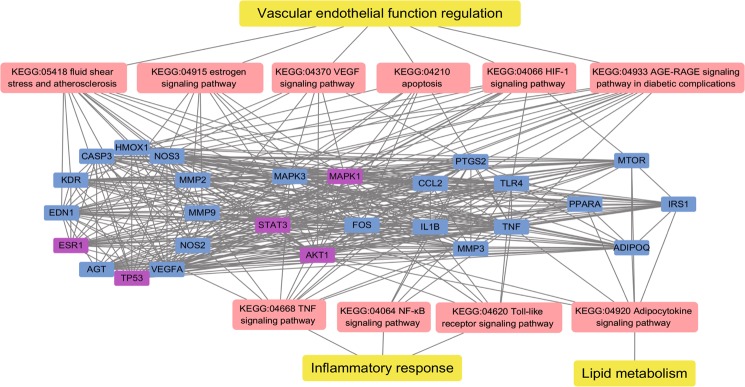
Core target-pathway network. Yellow nodes represent the functional modules. Pink nodes represent 10 signaling pathways from enrichment analysis of major targets. Blue nodes represent the important common targets from SMDOCH and CHD. Purple nodes represent the core common targets screened by the PPI network. Edges represent interactions among targets, signaling pathways and functional modules.

These clusters were subsequently input into DAVID and ClueGO for enrichment analysis, producing 100 BPs, 36 CCs, 34 MFs, and 98 KEGG pathways from eight clusters. Importantly, there were 10 signaling pathways overlapping with the common-target network in the three functional modules as the key mechanisms of SMDOCH on CHD. (1) Six of them were related to vascular endothelial function regulation, indicating that SMDOCH may regulate vascular endothelial function by affecting KEGG:04933, KEGG:05418, KEGG:04210, KEGG:04066, KEGG:04915, and KEGG:04370. These KEGG pathways were primarily associated with the common targets KDR, VEGFA, END1, NOS2, NOS3, AGT, ESR1, HMOX1, STAT3, TP53, CASP3, MMP2, and MMP9. (2) Three of them were related to inflammatory response, suggesting that SMDOCH may inhibit inflammatory response by affecting KEGG:04668, KEGG:04064, and KEGG:04620. These KEGG pathways were primarily associated with the common targets TNF, IL1B, TLR4, FOS, CCL2, and PTGS2. (3) One of them was related to lipid metabolism, indicating that SMDOCH may regulate lipid metabolism by affecting KEGG:04920. This KEGG pathway was primarily associated with the common targets ADIPOQ, MTOR, PPARA, and IRS1 (Fig. 4).

A PPI network was established to elucidate the link between SMDOCH-related targets, CHD-related targets, and other human proteins in the action mechanism of SMDOCH on CHD. As an extension of the common-target networks, we discovered new biological processes, which suggested that SMDOCH may also regulate vascular endothelial function through (KEGG:04014) ras signaling pathway, (KEGG:04110) cell cycle, and (KEGG:04115) p53 signaling pathway, which were related to cell proliferation, survival, growth, migration, differentiation, or cytoskeletal dynamism. SMDOCH may also regulate inflammatory response through (KEGG:04062) chemokine signaling pathway, which can provide directional cues for the trafficking and recruitment of leukocytes to the site of inflammation upon foreign insult. In addition, SMDOCH may also regulate lipid metabolism through the (KEGG:04910) insulin signaling pathway, which maintains lipid and glucose homeostasis. From data of GO terms, we found that SMDOCH may treat CHD by regulating gene repair and expression as well as macromolecular metabolism at the gene level with multiple synergies, such as (GO:0000398)mRNA splicing via spliceosome, (GO:0010467)gene expression, (GO:0010604)positive regulation of macromolecule metabolic process, and (GO:0010605)negative regulation of macromolecule metabolic process.

## Discussion

**Figure 5:**
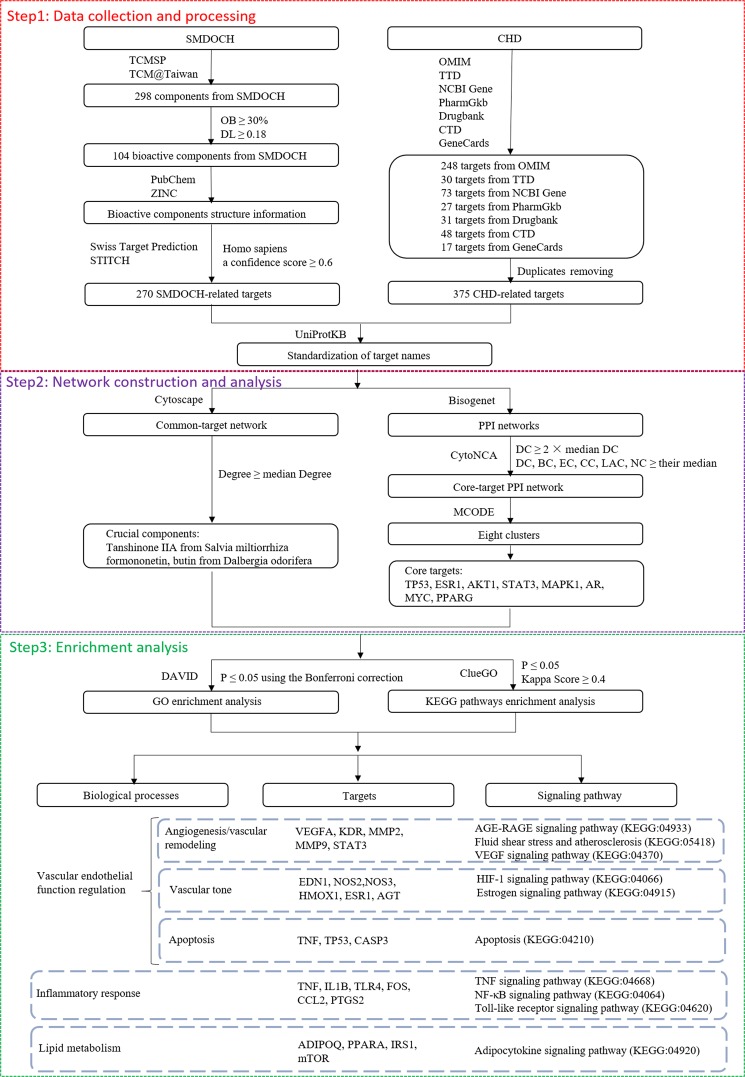
Diagram of the study design.

As a complex cardiovascular disease, CHD is a serious threat to the human health. At present, the commonly used chemical drugs mainly control the corresponding symptoms of CHD. TCM has characteristics of multi-component and multi-target, which can affect different biological processes to control symptoms and solve the fundamental problems. SMDOCH has been used for more than a thousand years to treat CHD^6–8^, but its unclear bioactive components and mechanisms greatly limit its clinical application. However, the relationships between bioactive components and TCM mechanisms have been explored by high-efficiency strategy of network pharmacology. Thus, in this study, we investigated the action mechanism of SMDOCH on CHD through network pharmacology using three aspects (Fig. 5).

Through GO and KEGG pathway enrichment analysis of common-target and PPI networks, 10 significant signaling pathways were screened as key action mechanisms of SMDOCH on CHD, and these pathways can be divided into three functional modules: ‘vascular endothelial function regulation’, ‘inflammatory response’, and ‘lipid metabolism’. Next, we used the KEGG Mapper to analyse the connections between upstream and downstream genes in the key signaling pathways.

The ‘Vascular endothelial function regulation’ model showed that SMDOCH may influence vascular endothelial function by regulating hormone (renin, angiotensin, oestrogen) activity and three key upstream pathways: KEGG:04933, KEGG:05418, and KEGG:04066. CHD is a heart disease caused by coronary artery atherosclerosis or organic stenosis and obstruction, resulting in myocardial ischemia, hypoxia, or necrosis^9^. Coronary artery stenosis disturbs blood flow with associated reciprocation, resulting in low-shear stress in the artery^10^. Myocardial ischemia-reperfusion will also lead to production of advanced glycation end products (AGEs)^11^. Through the KEGG:04933 and KEGG:05418 pathways, AGEs and low-shear stress activate intracellular signaling pathways that regulate five functions: firstly, AGEs and low-shear stress mediate the activation of PI3K-Akt and MAPK^12–14^, increasing the expression of activator protein 1 (AP-1) and NF-κB^15^, which activate pro-inflammatory cytokines, such as IL1B and TNF^16^, and various atherosclerosis-related genes, including CCL2^17^, MMP2, MMP9, VEGFA^18^, and END1^19^. Second, AGEs mediate the activation of the JAK-STAT pathway, leading to a cardioprotective response against ischemia, modulation of the cardiac microenvironment, and communication with cardiac fibroblasts^20^. Third, AGEs and low-shear stress mediate the activation of KEGG:04210, which eliminates damaged or redundant cells by activating CASP3 and CASP7, which are activated by the TNF and P53 signaling pathways^21^. Fourth, AGEs mediate the activation of AGT, which is closely related to extracellular fluid volume and blood pressure balance in the body^22^. Fifth, low-shear stress mediate the activation of KEGG:04370, which results in the expression of genes related to endothelial cell proliferation and migration, thereby promoting endothelial cell survival and improving vascular permeability^23^.

In addition, HIF-1 (KEGG:04066) expression is regulated by these targets of STAT3, TLR4, mTOR, MAPK1, and MAPK3. HIF-1 undergoes hydroxylation deactivation under normoxia, but becomes stable and interacts with coactivators under hypoxia. HIF-1- activation increases oxygen delivery through erythropoiesis, iron metabolism, angiogenesis (VEGFA), and vascular tone (END1, NOS2, NOS3, and HMOX1), as well as decreases oxygen consumption through promotion of anaerobic metabolism, inhibition of TCA cycle metabolism, and regulation of proliferation and apoptosis^24,25^. In addition, ESR1 (KEGG:04915) expression induces cardiomyocyte survival and vascular smooth muscle relaxation^26^.

The ‘Inflammatory response’ model showed that SMDOCH may influence inflammation response through three key signaling pathways: KEGG:04668, KEGG:04064, and KEGG:04620. Inflammatory response is a key factor leading to CHD^27^. Pro-inflammatory cytokines, such as TNF, IL1B, and TLR4, mediate the activation of MAPK, and then upregulate the expression of AP-1 and NF-κB, inducing the expression of pro-inflammatory cytokines and co-stimulatory molecules, including leukocyte recruitment (CCL2); activation of leukocytes; and synthesis of inflammatory cytokines (IL1B, TNF), transcription factors (FOS), and inflammatory mediators (PTGS2)^28–30^.

The ‘Lipid metabolism’ model showed that SMDOCH may influence lipid metabolism by regulating KEGG:04920. Abnormal glucose and lipid metabolisms are the main risk factors of CHD^31^. Adipocytokines, such as ADIPOQ and TNF, play an important role in regulating energy balance, metabolic homeostasis, and inflammation^32^. ADIPOQ not only increases the volume and number of adipocytes but also decreases plasma glucose and free fatty acids (FFAs)^33^. Through the KEGG:04920 pathway, ADIPOQ mediates the activation of the AMPK signaling pathway (KEGG:04152) and PPAR signaling pathway (KEGG:03320), which in turn regulates energy-consuming pathways, such as protein, glycogen, and fatty acid synthesis, as well as energy-producing pathways, such as glycolysis and fatty acid oxidation^34,35^. Some studies revealed that the proinflammatory factor TNF inhibits tyrosine phosphorylation of IRS1 by promoting the binding of IRS1 to serine, leading to insulin resistance^36^.

Recent studies have shown that some compounds in SM and DO have a certain effect on the body, confirming some of the biological processes found in this study. For instance, tanshinone IIA, the main bioactive component of SM, has many physiological functions, including endothelial protection, myocardial protection, vasodilation, apoptosis inhibition, reducing effect on vascular smooth muscle cells proliferation and migration (such as KEGG:04933, KEGG:05418, KEGG:04066), anti-inflammatory (such as KEGG:04668, KEGG:04064, KEGG:04620), and anti-atherosclerosis functions (such as KEGG:04920, KEGG:04152, and KEGG:03320)^37^. Formononetin in DO induces endothelial cell migration and dramatic actin cytoskeleton spatial modification through the ERα-enhanced-ROCK-II/MMP2/9 signaling pathways (such as KEGG:04915, KEGG:05418)^38^. In addition, butin in DO protects against ischemia/reperfusion-induced myocardial injury through the AMPK/Akt/GSK-3β/Nrf2 pathway (such as KEGG:04152)^39^.

SMDOCH may regulate these key signaling pathways and their genes directly to treat CDH. Moreover, SMDOCH may indirectly treat CHD through signaling pathways that do not overlap with the common-target network, such as KEGG:04014, KEGG:04110, KEGG:04115, KEGG:04062, and KEGG:04910. Further research is needed to verify or modify this finding.

However, there were some shortcomings in this study. First, the reliability of our investigation of potential targets of bioactive compounds using a similarity-based method was limited by the quality of the existing databases, and the target prediction methods cover only a few hundreds to thousands of targets that may introduce biases to the enrichment analysis. Second, network pharmacology is a virtual screening of potential bioactive components, targets, and pathways *in vitro*, which cannot reflect the real dynamic situation of drugs in the body. Third, the results can only be taken as hypotheses rather than solid conclusions proved by experiments. In the future, we strive to elucidate the potential bioactive components and pharmacological action mechanisms of SMDOCH on CHD *in vivo*.

In conclusion, the cardioprotective effect of the bioactive components of SMDOCH, such as tanshinone IIA from SM as well as formononetin and butin from DO, can be explained, at least in part, by the biological processes ‘vascular endothelial function regulation’, ‘inflammatory response’, and ‘lipid metabolism’ through the core targets TP53, ESR1, AKT1, STAT3, and MAPK1. In addition, 10 significant signaling pathways were screened to clarify the protective mechanism of SMDOCH on CHD. Six pathways belonged to the ‘vascular endothelial function regulation’ model, which primarily regulated hormone (renin, angiotensin, oestrogen) activity, and included three key upstream pathways that influence vascular endothelial function, namely KEGG:04933, KEGG:05418, and KEGG:04066. Three pathways, namely KEGG:04668, KEGG:04064, and KEGG:04620, belonged to the ‘inflammatory response’ model. One pathway (KEGG:04920) belonged to the ‘lipid metabolism’ model. To some extent, this study revealed the potential bioactive components and pharmacological mechanisms of SMDOCH on CHD, and provided a new direction for the development of new drugs for the treatment of CHD.

## Methods

### Data collection and processing

#### Composite components of SMDOCH

Data of the compounds of SMDOCH were mainly collected from two natural product databases for Chinese herbal medicines: TCMSP (http://ibts.hkbu.edu.hk/LSP/tcmsp.php, updated on May 31, 2014)^40^ and TCM@Taiwan (http://tcm.cmu.edu.tw, updated on Mar. 25, 2014)^41^ (Supplementary Table S1).

#### Screening of bioactive components

In clinical treatment, a TCM often used by oral administration. Oral bioavailability (OB)^42^ and drug-likeness (DL)^43^, two ADME-related models, are the main variables affecting the absorption of drugs by the gastrointestinal tract. Therefore, we screened bioactive components under the conditions of OB ≥ 30% and DL ≥ 0.18^44^ (Supplementary Table S2).

#### Target prediction of bioactive components

At present, many methods are available for target prediction, and these methods can be divided into four categories based on the prediction principles: 1) molecular docking-based methods, which are based on the three-dimensional (3D) structures of targets; 2) pharmacophore-based methods, which are based on structure and ligand pharmacophore mapping; 3) machine learning-based methods, which are based on databases; and 4) similarity-based methods, which are based on a prediction principle that similar drugs act on similar targets^45^. In this study, we selected two similarity-based webservers, Swiss Target Prediction (http://www.swisstargetprediction.ch/)^46^ and STITCH (http://stitch.embl.de/)^47^, to predict the target of TCM components. Considering that the chemical–protein associations integrated in these databases are from pathway and experimental databases as well as from the literature, each proposed interaction can be traced back to the original data sources.

First, we obtained information on the structure of the bioactive components, including molecular structures, canonical smiles, and their ‘sdf’ files from the product databases of PubChem (https://pubchem.ncbi.nlm.nih.gov/, updated on Jan. 1, 2019)^48^ and ZINC (http://zinc15.docking.org, updated on Feb. 14, 2018)^49^. Next, we predicted the target of bioactive components using public databases, namely Swiss Target Prediction and STITCH, with the species limited to *‘Homo sapiens’* and a confidence score ≥0.6. Finally, we standardized the target names using UniProtKB (https://www.uniprot.org/)^50^ (Supplementary Table S3).

#### CHD-related targets

After removing duplicates and standardizing the target names using UniProtKB, CHD-related targets were collected from seven databases to investigate the relationship between targets and diseases from different perspectives (Supplementary Table S4). Taking advantage of the different characteristics of each database, we selected different keywords and criteria to search CHD-related targets. ‘Coronary heart disease’ was used as a keyword to search the Drugbank database (https://www.drugbank.ca, updated on Dec. 20, 2018)^51^ and the National Centre for Biotechnology Information Gene (NCBI Gene, https://www.ncbi.nlm.nih.gov/gene/, updated on Jan. 4, 2019)^52^. Next, 31 and 73 genes were obtained from the two databases, respectively. ‘Coronary heart disease’, ‘Coronary artery disease’, ‘Acute coronary syndrome’, ‘Coronary artery restenosis’, ‘Coronary disorder diagnosis’, and ‘Myocardial infarction’ were used as keywords to search the Therapeutic Target Database (TTD, http://bidd.nus.edu.sg/group/cjttd/, updated on Sep. 15, 2017)^53^, and 30 genes were obtained. ‘Coronary’ was used as a keyword to search the Online Mendelian Inheritance in Man (OMIM, http://omim.org/, updated on Jan. 15, 2019)^54^, and 467 pieces of information were obtained, including 248 genes. ‘Coronary Disease’ was used as a keyword to search the Pharmacogenomics Knowledge Implementation (PharmGkb, https://www.pharmgkb.org/, updated on Jan. 11, 2019)^55^, Comparative Toxicogenomics Database (CTD, http://ctdbase.org/, updated on Jan. 5, 2019)^56^, and GeneCards (https://www.genecards.org/, updated on Jan. 31, 2019)^57^. A total of 27 genes were obtained from PharmGkb, 48 genes were obtained from CTD with a screening criteria of inference score ≥60, and 17 genes were obtained from GeneCards with a screening criteria of relevance score ≥60.

### Network construction

#### Common-target network construction

After screening the common targets from SMDOCH and CHD (Supplementary Table S5), we constructed a common-target network by Cytoscape (http://www.cytoscape.org, version 3.7.1)^58^.

#### PPI network construction

Maintaining cellular homeostasis requires a combination of proteins with other molecules, such as genes, small compounds, and other proteins. A PPI network was established to elucidate the link between predicted targets and other human proteins, and it has emerged as a promising method for drug discovery^59^. A PPI network can be built and visually analysed at different levels of details by Bisogenet, a plug-in of Cytoscape, which contains six main PPI databases: Molecular INTeraction Database (MINT), IntAct Molecular Interaction Database (IntAct), Database of Interacting Proteins (DIP), Human Protein Reference Database (HPRD), Biomolecular Interaction Network Database (BIND), and Biological General Repository for Interaction Datasets (BioGRID)^60^. In the Bisogenet program, we defined the identifiers as ‘Homo sapiens, proteins identifiers only’, selected the data sources of PPI, and finally set the distance from the input set to new nodes as ‘1’ and the representation for the output as ‘proteins’. Next, PPI networks of CHD-related targets and SMDOCH-related targets were constructed.

#### Central network evaluation

With the rapid progress in bioinformatics technology, central network evaluation has become the primary method for screening core proteins in PPI networks containing large amounts of genomic and proteomic data. First, we merged the PPI networks of CHD-related targets and SMDOCH-related targets to acquire an intersection. Next, we used CytoNCA, a plug-in of Cytoscape, to evaluate the intersection. Six centrality measures provided by CytoNCA were used to filter the data, including degree centrality (DC), closeness centrality (CC), network centrality (NC), betweenness centrality (BC), local average connectivity-based method (LAC), and eigenvector centrality (EC)^61^. First, the data were preliminarily processed by the screening criteria of ‘DC ≥ 2 × median DC’, and then secondarily screened as core targets by the screening criteria of ‘DC, BC, EC, CC, LAC, and NC greater than or equal to their median’^62^ (Supplementary Table S6).

#### Cluster analysis

Cluster analysis is used for the sub-regional analysis of complex PPI networks, which can extract nodes with the same or similar attributes as a cluster^63^. In this study, we chose MCODE^64^, a cluster analysis algorithm in Cytoscape, to conduct cluster analysis for the core-target PPI network (Supplementary Table S7).

### Enrichment analysis

We used the Database for Annotation Visualization and Integrated Discovery (DAVID, https://david.nicifcrf.gov/, version 6.8) for gene ontology (GO) enrichment analysis^65,66^ with the screening criteria of P ≤ 0.05 using the Bonferroni correction^67^. The Bonferroni correction compensates for multiple comparisons by dividing the level of significance by the number of comparisons^68^. To uncover functionally grouped gene pathway annotation networks, we used ClueGO, a plug-in of Cytoscape, to apply Kyoto Encyclopaedia of Genes and Genomes (KEGG) pathways enrichment analysis with P ≤ 0.05 and Kappa Score ≥0.4 as screening criteria^69^. Moreover, we used the KEGG Mapper (https://www.genome.jp/kegg/mapper.html), a collection of tools for ‘Search Pathway’ and ‘Color Pathway’, to analyse the connections between upstream and downstream genes in key signaling pathways^70–72^. Next, we performed enrichment analysis for common-target networks and PPI networks to elucidate the action mechanism of SMDOCH in the treatment of CHD (Supplementary Tables S8–10).

## Supplementary information

Supplementary Information

Supplementary Table S1

Supplementary Table S2

Supplementary Table S3

Supplementary Table S4

Supplementary Table S5

Supplementary Table S6

Supplementary Table S7

Supplementary Table S8

Supplementary Table S9

Supplementary Table S10

## Data Availability

All data generated or analysed during this study are included in this published article and its Supplementary Information Files.

## References

[1] Neumann F (2019). 2018 ESC/EACTS Guidelines On Myocardial Revascularization. Eur. Heart J..

[2] Wong ND (2014). Epidemiological Studies of CHD and the Evolution of Preventive Cardiology. Nat. Rev. Cardiol..

[3] Doenst T (2019). PCI and CABG for Treating Stable Coronary Artery Disease. J. Am. Coll. Cardiol..

[4] Hopkins AL (2007). Network Pharmacology. Nat. Biotechnol..

[5] Musunuru K, Kathiresan S (2019). Genetics of Common, Complex Coronary Artery Disease. Cell..

[6] Mu F (2017). Metabonomic Strategy for the Evaluation of Chinese Medicine Salvia miltiorrhiza and Dalbergia odorifera Interfering with Myocardial Ischemia/Reperfusion Injury in Rats. Rejuvenation Res..

[7] Sugiyama A, Zhu BM, Takahara A, Satoh Y, Hashimoto K (2002). Cardiac Effects of Salvia Miltiorrhiza/Dalbergia Odorifera Mixture, an Intravenously Applicable Chinese Medicine Widely Used for Patients with Ischemic Heart Disease in China. Circ. J..

[8] Lin R (2018). Cardioprotective Effects and Underlying Mechanism of Radix Salvia Miltiorrhiza and Lignum Dalbergia Odorifera in a Pig Chronic Myocardial Ischemia Model. Int. J. Mol. Med..

[9] Henderson A (1996). Coronary Heart Disease: Overview. Lancet..

[10] Koskinas KC, Chatzizisis YS, Antoniadis AP, Giannoglou GD (2012). Role of Endothelial Shear Stress in Stent Restenosis and Thrombosis: Pathophysiologic Mechanisms and Implications for Clinical Translation. J. Am. Coll. Cardiol..

[11] Bucciarelli LG (2006). Receptor for Advanced-Glycation End Products: Key Modulator of Myocardial Ischemic Injury. Circulation..

[12] Zhao LM, Zhang W, Wang LP, Li GR, Deng XL (2012). Advanced Glycation End Products Promote Proliferation of Cardiac Fibroblasts by Upregulation of KCa3.1 Channels. Pflugers Arch..

[13] Melchior B, Frangos JA (2014). Distinctive Subcellular Akt-1 Responses to Shear Stress in Endothelial Cells. J. Cell. Biochem..

[14] Surapisitchat J (2001). Fluid Shear Stress Inhibits TNF-alpha Activation of JNK but Not ERK1/2 Or P38 in Human Umbilical Vein Endothelial Cells: Inhibitory Crosstalk Among MAPK Family Members. Proc Natl Acad Sci USA.

[15] Hsieh HL (2008). Sphingosine 1-Phosphate Induces EGFR Expression Via Akt/NF-kappaB and ERK/AP-1 Pathways in Rat Vascular Smooth Muscle Cells. J. Cell. Biochem..

[16] Ma S, Bai Z, Wu H, Wang W (2019). The DPP-4 Inhibitor Saxagliptin Ameliorates ox-LDL-induced Endothelial Dysfunction by Regulating AP-1 and NF-kappaB. Eur. J. Pharmacol..

[17] Wang N (2019). Hyaluronic Acid Oligosaccharides Improve Myocardial Function Reconstruction and Angiogenesis against Myocardial Infarction by Regulation of Macrophages. Theranostics..

[18] Adya R, Tan BK, Punn A, Chen J, Randeva HS (2008). Visfatin Induces Human Endothelial VEGF and MMP-2/9 Production Via MAPK and PI3K/Akt signaling Pathways: Novel Insights Into Visfatin-Induced Angiogenesis. Cardiovasc. Res..

[19] Adamopoulos C (2016). Advanced Glycation End Products Upregulate Lysyl Oxidase and Endothelin-1 in Human Aortic Endothelial Cells Via Parallel Activation of ERK1/2-NF-kappaB and JNK-AP-1 Signaling Pathways. Cell. Mol. Life Sci..

[20] O Sullivan, K. E., Breen, E. P., Gallagher, H. C., Buggy, D. J. & Hurley, J. P. Understanding STAT3 Signaling in Cardiac Ischemia. *Basic Res. Cardiol*. **111** (2016). 10.1007/s00395-016-0543-8 27017613

[21] Wang X (2014). MicroRNA-125b Protects Against Myocardial Ischaemia/Reperfusion Injury Via Targeting P53-Mediated Apoptotic signaling and TRAF6. Cardiovasc. Res..

[22] Cheng CL (2012). Advanced Glycation End-Products Activate the Renin-Angiotensin System through the RAGE/PI3-K Signaling Pathway in Podocytes. Clin. Invest. Med..

[23] Heinolainen K (2017). VEGFR3 Modulates Vascular Permeability by Controlling VEGF/VEGFR2 Signaling. Circ. Res..

[24] Semenza GL (2014). Hypoxia-Inducible Factor 1 and Cardiovascular Disease. Annu. Rev. Physiol..

[25] Ong SG, Hausenloy DJ (2012). Hypoxia-Inducible Factor as a Therapeutic Target for Cardioprotection. Pharmacol Ther..

[26] Mendelsohn ME, Karas RH (1999). The Protective Effects of Estrogen On the Cardiovascular System. N Engl J Med..

[27] Kaptoge S (2014). Inflammatory Cytokines and Risk of Coronary Heart Disease: New Prospective Study and Updated Meta-Analysis. Eur. Heart J..

[28] Chen YM (2004). Dual Regulation of Tumor Necrosis Factor-Alpha-Induced CCL2/monocyte Chemoattractant Protein-1 Expression in Vascular Smooth Muscle Cells by Nuclear factor-kappaB and Activator Protein-1: Modulation by Type III Phosphodiesterase Inhibition. J. Pharmacol. Exp. Ther..

[29] Smolinska MJ, Page TH, Urbaniak AM, Mutch BE, Horwood NJ (2011). Hck Tyrosine Kinase Regulates TLR4-induced TNF and IL-6 Production Via AP-1. J. Immunol..

[30] Said FA (2002). TNF-alpha, Inefficient by Itself, Potentiates IL-1beta-induced PGHS-2 Expression in Human Pulmonary Microvascular Endothelial Cells: Requirement of NF-kappaB and P38 MAPK Pathways. Br J Pharmacol..

[31] DiNicolantonio JJ, Lucan SC, O’Keefe JH (2016). The Evidence for Saturated Fat and for Sugar Related to Coronary Heart Disease. Prog. Cardiovasc. Dis..

[32] Sook Lee E (2013). Association Between Adiponectin Levels and Coronary Heart Disease and Mortality: A Systematic Review and Meta-Analysis. Int. J. Epidemiol..

[33] Wang Y, Ma XL, Lau WB (2017). Cardiovascular Adiponectin Resistance: The Critical Role of Adiponectin Receptor Modification. Trends Endocrinol Metab..

[34] Kohan AB, Talukdar I, Walsh CM, Salati LM (2009). A Role for AMPK in the Inhibition of Glucose-6-Phosphate Dehydrogenase by Polyunsaturated Fatty Acids. Biochem. Bioph. Res. Co..

[35] Antonopoulos AS (2016). Mutual Regulation of Epicardial Adipose Tissue and Myocardial Redox State by PPAR-gamma/Adiponectin signaling. Circ. Res..

[36] Pirola L, Johnston AM, Van Obberghen E (2004). Modulation of Insulin Action. Diabetologia..

[37] Ren J, Fu L, Nile SH, Zhang J, Kai G (2019). Salvia Miltiorrhiza in Treating Cardiovascular Diseases: A Review On its Pharmacological and Clinical Applications. Front Pharmacol..

[38] Li S (2015). Formononetin Promotes Angiogenesis through the Estrogen Receptor Alpha-Enhanced ROCK Pathway. Sci Rep..

[39] Duan J (2017). Protective Effect of Butin Against Ischemia/Reperfusion-Induced Myocardial Injury in Diabetic Mice: Involvement of the AMPK/GSK-3beta/Nrf2 Signaling Pathway. Sci Rep..

[40] Ru J (2014). TCMSP: A Database of Systems Pharmacology for Drug Discovery From Herbal Medicines. J Cheminform..

[41] Chen CY (2011). TCM Database@Taiwan: The World’s Largest Traditional Chinese Medicine Database for Drug Screening in Silico. PLoS One..

[42] Xu X (2012). A Novel Chemometric Method for the Prediction of Human Oral Bioavailability. Int. J. Mol. Sci..

[43] Walters WP, Murcko MA (2002). Prediction of ‘Drug-Likeness’. Adv. Drug Deliver. Rev..

[44] Feng, W., Ao, H., Yue, S. & Peng, C. Systems Pharmacology Reveals the Unique Mechanism Features of Shenzhu Capsule for Treatment of Ulcerative Colitis in Comparison with Synthetic Drugs. *Sci. Rep.-UK*. **8** (2018). 10.1038/s41598-018-34509-1 PMC6212405 30385774

[45] Wu, Z., Li, W., Liu, G. & Tang, Y. Network-Based Methods for Prediction of Drug-Target Interactions. *Frontiers in Pharmacology*. **9** (2018). 10.3389/fphar.2018.01134 PMC6189482 30356768

[46] Gfeller D (2014). SwissTargetPrediction: A Web Server for Target Prediction of Bioactive Small Molecules. Nucleic Acids Res..

[47] Kuhn M, von Mering C, Campillos M, Jensen LJ, Bork P (2007). STITCH: Interaction Networks of Chemicals and Proteins. Nucleic Acids Res..

[48] Kim S (2019). PubChem 2019 Update: Improved Access to Chemical Data. Nucleic Acids Res..

[49] Sterling T, Irwin JJ (2015). ZINC 15 – Ligand Discovery for Everyone. J. Chem. Inf. Model..

[50] Consortium TU (2015). UniProt: A Hub for Protein Information. Nucleic Acids Res..

[51] Wishart DS (2006). DrugBank: A Comprehensive Resource for in Silico Drug Discovery and Exploration. Nucleic Acids Res..

[52] Brown GR (2015). Gene: A Gene-Centered Information Resource at NCBI. Nucleic Acids Res..

[53] Li YH (2018). Therapeutic Target Database Update 2018: Enriched Resource for Facilitating Bench-To-Clinic Research of Targeted Therapeutics. Nucleic Acids Res..

[54] Amberger JS, Hamosh A (2017). Searching Online Mendelian Inheritance in Man (OMIM): A Knowledgebase of Human Genes and Genetic Phenotypes. Curr Protoc Bioinformatics..

[55] Whirl-Carrillo M (2012). Pharmacogenomics Knowledge for Personalized Medicine. Clin. Pharmacol. Ther..

[56] Davis AP (2019). The Comparative Toxicogenomics Database: Update 2019. Nucleic Acids Res..

[57] Stelzer G (2016). The GeneCards Suite: From Gene Data Mining to Disease Genome Sequence Analyses. Curr Protoc Bioinformatics..

[58] Shannon P (2003). Cytoscape: A Software Environment for Integrated Models of Biomolecular Interaction Networks. Genome Res..

[59] Murakami Y, Tripathi LP, Prathipati P, Mizuguchi K (2017). Network Analysis and in Silico Prediction of Protein-Protein Interactions with Applications in Drug Discovery. Curr Opin Struct Biol..

[60] Martin A (2010). BisoGenet: A New Tool for Gene Network Building, Visualization and Analysis. BMC Bioinformatics..

[61] Tang Y, Li M, Wang J, Pan Y, Wu F (2015). CytoNCA: A Cytoscape Plugin for Centrality Analysis and Evaluation of Protein Interaction Networks. Biosystems..

[62] Song, W., Ni, S., Fu, Y. & Wang, Y. Uncovering the Mechanism of Maxing Ganshi Decoction On Asthma From a Systematic Perspective: A Network Pharmacology Study. *Sci. Rep.-UK*. **8** (2018). 10.1038/s41598-018-35791-9 PMC6255815 30478434

[63] Ahmed HA, Bhattacharyya DK, Kalita JK (2015). Core and Peripheral Connectivity Based Cluster Analysis Over PPI Network. Comput. Biol. Chem..

[64] Bader GD, Hogue CW (2003). An Automated Method for Finding Molecular Complexes in Large Protein Interaction Networks. BMC Bioinformatics..

[65] Huang DW, Sherman BT, Lempicki RA (2009). Systematic and Integrative Analysis of Large Gene Lists Using DAVID Bioinformatics Resources. Nat. Protoc..

[66] Huang DW, Sherman BT, Lempicki RA (2009). Bioinformatics Enrichment Tools: Paths Toward the Comprehensive Functional Analysis of Large Gene Lists. Nucleic Acids Res..

[67] Chen H (2013). Gene Expression Alterations in Bipolar Disorder Postmortem Brains. Bipolar Disord..

[68] Etymologia: Bonferroni Correction. *Emerg. Infect. Dis*. **21**, 289 (2015). 10.3201/eid2102.ET2102 PMC4313667 25786274

[69] Bindea G (2009). ClueGO: A Cytoscape Plug-In to Decipher Functionally Grouped Gene Ontology and Pathway Annotation Networks. Bioinformatics..

[70] Kanehisa, M. Toward Understanding the Origin and Evolution of Cellular Organisms. *Protein Sci*. (2019). 10.1002/pro.3715 PMC6798127 31441146

[71] Kanehisa M, Goto S (2000). KEGG: Kyoto Encyclopedia of Genes and Genomes. Nucleic Acids Res..

[72] Kanehisa M, Sato Y, Furumichi M, Morishima K, Tanabe M (2019). New Approach for Understanding Genome Variations in KEGG. Nucleic Acids Res..

