# Supplementary material for: A network pharmacology approach to reveal the protective mechanism of Salvia miltiorrhiza-Dalbergia odorifera coupled-herbs on coronary heart disease
Source: Sci Rep. 2019 Dec 18;9:19343. doi: 10.1038/s41598-019-56050-5 (PMC6920415; doi:10.1038/s41598-019-56050-5)
Supplement: Supplementary file 1 — Supplementary Information [file 41598_2019_56050_MOESM1_ESM.docx]

***Supplementary Information***

**A** **network pharmacology approach to reveal the** **protective mechanism of** **Salvia miltiorrhiza-****Dalbergia odorifera coupled-herbs on** **coronary heart disease**

**Fei Li^1,2, +^, Jialin Duan^1, +^, Meina Zhao^1,4, +^,** **Shaojie Huang^1, +^, Fei Mu^1^, Jing Su^4^, Kedi Liu^4^, Yang Pan^3^, Xinming Lu^6^, Jing Li^6^,** **Peifeng Wei^4, *^,** **Miaomiao Xi^1,5, *^, Aidong Wen^1, *^**

^1^Department of Pharmacy, Xijing Hospital, Fourth Military Medical University, Xi'an, Shaanxi, 710032, China;

^2^Department of Pharmacy, The Hospital of 92012 Troops, PLA Navy, Zhoushan, Zhejiang, 316000, China;

^3^Department of Chinese Materia Medical and Natural Medicines, School of Pharmacy, Fourth Military Medical University, Xi'an, Shaanxi, 710032, China;

^4^College of Pharmacy, Shaanxi University of Chinese Medicine, Xianyang, Shaanxi, 712046, China;

^5^TANK Medicinal Biology Institute of Xi'an, Xi'an, Shaanxi, 710032, China;

^6^YouYi Clinical Laboratories of Shaanxi, Xi'an, Shaanxi, 710032, China.

^*^Corresponding authors. Prof. Wei is contacted at College of Pharmacy, Shaanxi University of Chinese Medicine, Century Avenue, Qindu District, Xianyang, Shaanxi, China; Prof. Wen and Prof. Xi both are contacted at Changle West Road 127, Xi’an, Shaanxi, China.

Email address: [weipeifeng@163.com](mailto:weipeifeng@163.com) (PF. Wei), aidongwen2015@163.com (AD. Wen), miaomiaoxi2014@163.com (MM. Xi).

^+^These authors contributed equally to this work.

**1 Supplementary Tables**

**Supplementary Table S1** Components of each herb in SMDOCH.

**Supplementary Table S2** Bioactive components of each herb in SMDOCH.

**Supplementary Table S3** Potential targets of bioactive components in SMDOCH.

**Supplementary Table S4** Known CHD-related targets.

**Supplementary Table S5** 58 common targets between SMDOCH and CHD.

**Supplementary Table S6** Network centrality analysis and evaluation.

**Supplementary Table S7** The Cluster of the core-target PPI network.

**Supplementary Table S8** GO and KEGG pathway analysis for the 58 common targets.

**Supplementary Table S9** GO analysis for each cluster.

**Supplementary Table S10** KEGG pathway analysis for each cluster.
